# Neurochemical and molecular mechanisms underlying the retrieval-extinction effect

**DOI:** 10.1007/s00213-018-5121-3

**Published:** 2019-01-17

**Authors:** Emma N. Cahill, Amy L. Milton

**Affiliations:** 1 Department of Physiology, Development and Neuroscience, University of Cambridge, Downing Site, Cambridge, CB2 3EG UK; 2 Department of Psychology, University of Cambridge, Downing Site, Cambridge, CB2 3EB UK; 3 Behavioural and Clinical Neuroscience Institute, Cambridge, CB2 3EB UK

**Keywords:** Extinction, Reconsolidation, Retrieval-Extinction, Memory, Behaviour

## Abstract

Extinction within the reconsolidation window, or ‘retrieval-extinction’, has received much research interest as a possible technique for targeting the reconsolidation of maladaptive memories with a behavioural intervention. However, it remains to be determined whether the retrieval-extinction effect—a long-term reduction in fear behaviour, which appears resistant to spontaneous recovery, renewal and reinstatement—depends specifically on destabilisation of the original memory (the ‘reconsolidation-update’ account) or represents facilitation of an extinction memory (the ‘extinction-facilitation’ account). We propose that comparing the neurotransmitter systems, receptors and intracellular signalling pathways recruited by reconsolidation, extinction and retrieval-extinction will provide a way of distinguishing between these accounts.

## Introduction

Since its first description by Monfils et al. (2009), the ‘extinction within the reconsolidation window’ phenomenon has received intense research attention. Originally described in rats that had undergone auditory cued pavlovian fear conditioning 24 h prior to the manipulation, extinction within the reconsolidation window, or ‘retrieval-extinction’, involves a brief re-exposure to the conditioned stimulus (CS) previously associated with an aversive outcome (i.e. retrieval), and shortly afterwards repeated re-exposure to the CS without the aversive outcome (i.e. extinction). In contrast to ‘standard’ extinction, where there is a reduction in the expression of the fear memory that returns with changes in temporal or spatial context (Bouton 1988), retrieval-extinction was originally described as producing a long-term reduction in fear that does not show spontaneous recovery, renewal or reinstatement (Monfils et al. 2009). Thus, it has been hypothesised (Monfils et al. 2009; Tedesco et al. 2014) that retrieval-extinction induces the updating of the original ‘CS-fear’ memory to a ‘CS-no-fear’ memory, rather than the acquisition of a novel, inhibitory CS-no-fear memory competing with the CS-fear memory for behavioural expression, as has been hypothesised to be the case with standard extinction training.

At the heart of this protocol is the notion of conducting extinction training within a time window of memory destabilisation. As noted above, the standard view is that retrieval-extinction induces updating of the original CS-US memory through reconsolidation mechanisms. If this is the case, then it would be predicted that the retrieval-extinction effect should depend critically upon reactivation and destabilisation of the original memory. An alternative view is that the retrieval-extinction procedure instead leads to enhancement of extinction, perhaps through facilitation of standard extinction mechanisms, or by reducing the context-specificity of extinction (Bouton 1988). Psychologically, it has been difficult to distinguish between these two hypotheses, particularly due to the effect being relatively fragile and not always replicable (Auber et al. 2013). This was most dramatically demonstrated by a lack of replication in a recent pre-registered study (Luyten and Beckers 2017). Indeed, different levels of success with this procedure have been reported for both aversive and appetitive memories (Kredlow et al. 2016; Hutton-Bedbrook and McNally 2013). Based on the literature, it remains debatable whether the retrieval-extinction effect is a reproducible across different tasks. By contrast, evidence has accumulated on the molecular requirements of extinction and reconsolidation. We suggest that by analysing the molecules and signalling pathways required to be activated for the retrieval-extinction effect to occur, it may be possible to determine whether the phenomenon reflects ‘overwriting’ of the original memory (the ‘reconsolidation-update’ account) or an enhanced form of extinction. Therefore, we focus this review on comparing the neurochemical and intracellular signalling mechanisms shown to be required for fear memory reconsolidation or extinction, in order to discern whether a reconsolidation or extinction explanation can best account for the findings obtained by extinguishing a memory within the reconsolidation window.

Here, we structure our analysis by focusing on the requirement for neurochemical systems previously implicated in fear mnemonic processes. We focus on pavlovian fear conditioning tasks in rodents, with some rare relevant exceptions, because pavlovian fear conditioning and extinction are particularly well-characterised psychologically and neurobiologically. The focus on animal, rather than human, research is due to the more direct causal manipulations that are possible and ethical in animals. Furthermore, both fear conditioning and extinction can occur within single training sessions, allowing for the neurochemical and intracellular signalling changes associated with these processes to be more readily assessed with temporal precision. We focus primarily on mechanisms within the basolateral amygdala—an area critically involved in the storage, retrieval and reconsolidation of fear memories (Nader et al. 2000; Fanselow and Gale 2003)—but also on the hippocampus due to its critical role in contextual fear memory, and the infralimbic and prelimbic cortices for their important roles in fear memory extinction, although undoubtedly, other sites such as the periaqueductal grey are also important for fear memory processing (see McNally et al. 2011). Beginning with signalling at the cell-surface level, we review the neurotransmitters, second messengers, third messengers and transcription factors that have been implicated in the retrieval-extinction effect, and by comparing with the mechanisms required for memory updating and extinction per se, we consider whether the retrieval-extinction effect is more consistent with memory updating or a facilitation of extinction.

## Extinction within the reconsolidation window as a psychological phenomenon: some key definitions

Before reviewing the neurochemical and molecular mechanisms implicated in the retrieval-extinction effect, it is important to consider the psychological processes that might be engaged by the retrieval-extinction technique. Specifically, it is important to clarify what is meant by both the ‘retrieval’ and ‘reactivation’ of a memory, as these terms have not always been consistently used in the literature. In some instances, retrieval has been used to refer to the *expression* of behaviour, which is used to confirm that the memory exists, at least in animal studies—for example, conditioned freezing is used as an index of the strength of a conditioned fear memory in rats. Reactivation is sometimes used interchangeably with retrieval, but here, we use this term to refer specifically to the procedure used to induce the reconsolidation of a previously consolidated memory. Reconsolidation is a multi-step process in which the memory is first ‘reactivated’ (or, more mechanistically, destabilised) into a labile, malleable ‘active’ state before undergoing ‘reconsolidation’ (or restabilisation) back into a stable, persistent ‘inactive’ state, possibly in an updated or modified form (Lewis 1979; Nader 2003). While the procedures used in the laboratory to reactivate a memory often also result in retrieval, reactivation and retrieval are not synonymous and are doubly dissociable (Rodriguez-Ortiz et al. 2012; Sevenster et al. 2012; Barreiro et al. 2013; Otis et al. 2013; Milton et al. 2013).

By contrast to reconsolidation, extinction of a behavioural response is widely accepted to require new learning (see Dunsmoor et al. 2015, for review). Both pavlovian memory reactivation and pavlovian extinction can be induced by non-reinforced re-exposure to the previously trained conditioned stimulus, with the dominant mnemonic process being determined by the extent of re-exposure. Specifically, brief re-exposure tends to bias towards the engagement of reconsolidation mechanisms, while prolonged re-exposure promotes the formation of a new extinction memory (Lee et al. 2006; Merlo et al. 2014). Though both reconsolidation and extinction can be induced by varying degrees of re-exposure to the conditioned stimulus, the mechanisms underlying both processes, and the transition between them, are yet to be fully characterised (Merlo et al. 2014; Merlo et al. 2018).

The mainstream view of the retrieval-extinction effect makes the assumption that the original memory is destabilised during a brief re-exposure session, and while the memory is unstable, it is updated to indicate that the conditioned stimulus no longer predicts the aversive outcome. Thus, rather than forming a new inhibitory memory, it is postulated (Monfils et al. 2009; Schiller et al. 2010) that the previous fear memory is overwritten during its reconsolidation. However, hereafter, we refer to retrieval-extinction, describing the procedure, rather than extinction within the reconsolidation window, which is a more mechanistically loaded term. Thus, if the retrieval-extinction effect depends upon reconsolidation, then reactivation/destabilisation of the memory should be required in order to observe the subsequent enhanced attenuation of fear associated with this technique, and it would be predicted that the neurochemical and intracellular signalling mechanisms required for memory destabilisation would also be recruited during the retrieval-extinction procedure.

## Signalling at the cell surface: neurotransmitters and receptors required for (retrieval-)extinction

There have been considerable efforts to characterise the neurotransmitters required for the initial consolidation of a fear memory and its subsequent extinction, but to the best of our knowledge, there have been no studies performed to directly measure (e.g. through microdialysis or voltammetry) how neurotransmitter levels are regulated by the combination of memory retrieval and extinction in quick succession, or by memory destabilisation itself. However, considering the strong evidence that destabilisation is induced when there is a ‘violation of expectations’ between what an organism expects and what actually occurs (Pedreira et al. 2004), more mechanistically characterised as ‘prediction error’, it is perhaps surprising that there are no studies attempting to characterise changes in dopaminergic signalling during fear memory reactivation. However, this may reflect on the ongoing debate regarding the requirement for dopamine in signalling aversive outcomes (for review see Ilango et al. 2012). One might predict, based on understanding of reward prediction errors (Schultz et al. 1997) and the capacity of dopamine to distinctly modulate synaptic plasticity in a number of brain regions (Jay 2003), that memory destabilisation may recruit changes in levels of dopamine in the areas that undergo memory-dependent plasticity (i.e. the amygdala or hippocampus). Despite this, the existing literature has built a picture of the changing flux of neurotransmitters such as dopamine (DA), noradrenaline (NA), serotonin (5-HT), gamma-aminobutyrinic acid (GABA) and glutamate across key brain regions during the retrieval and extinction of fear memories.

### Correlative studies of neurotransmitter changes during fear memory retrieval and extinction

The retrieval of fear memory correlates with increased dopamine and serotonin signalling in the amygdala (Yokoyama et al. 2005), a key locus for fear conditioning (Weiskrantz 1956; Wilensky et al. 2006). Surprisingly, considering that activation of the AMPA-subtype of glutamate receptor is required for fear memory retrieval (see below), increases in glutamatergic signalling have not been observed during fear memory retrieval, though levels do increase during the initial acquisition of pavlovian fear memory (Yokoyama et al. 2005). This may reflect the limitations of microdialysis techniques in detecting small changes in glutamate release (Yokoyama et al. 2005). Other studies have focused on the wider network of structures supporting fear memories, with reports showing increased extracellular dopamine and noradrenaline levels in the medial prefrontal cortex (mPFC) when a fear memory is retrieved 2–3 h post-acquisition (Feenstra et al. 2001). Thus, fear memory retrieval is correlated with increased dopaminergic and serotonergic signalling in the amygdala and increased dopaminergic and noradrenergic signalling in the mPFC.

Neurotransmitter levels have been measured in both the amygdala and the mPFC during extinction of fear memory. During tests of long-term memory (more than a week since fear conditioning), ten unreinforced CS presentations, usually sufficient to produce extinction of the fear memory (Lee et al. 2006; Merlo et al. 2014), were observed to rapidly and persistently depress levels of GABA within the basolateral amygdala (BLA) (Stork et al. 2002). However, the lack of behavioural data in this study makes it unclear whether the depression of GABAergic signalling was purely an effect of retrieval, or reflected extinction. During extinction training, there are increases in DA and NA levels in the mPFC (Hugues et al. 2007), and it appears that the increase in DA in the mPFC is associated with greater reductions in fear following extinction training. It has been suggested that DA increases observed in the mPFC may be linked to novelty, including the presentation of unpaired stimuli (CS^−^) or familiar stimuli (CS^+^) in a novel context, at the start of extinction training (Wilkinson et al. 1998). Certainly, presentation of a surprising but neutral outcome in association with a previously fear-conditioned cue reduces the likelihood of spontaneous recovery, as compared to standard extinction training (Dunsmoor et al. 2015). Furthermore, a surprising presentation of weak shock in a novel context produces a similar attenuation of subsequent fear expression as does brief re-exposure to the training context prior to extinction training, preventing reinstatement and spontaneous recovery of contextual fear memory (Liu et al. 2014). It is unclear from these data, however, whether the US presentation was *required* for the subsequent reduction in spontaneous recovery, or whether exposure to the novel context alone would have been sufficient to potentiate extinction learning, as was noted in similar studies (de Carvalho Myskiw et al. 2014). If the latter, then it may be that a common feature of successful retrieval-extinction manipulations is the use of retrieval stimuli (whether related to the original training experience or not) that trigger increased DA in the mPFC. Causal studies, pharmacologically enhancing DA levels using L-DOPA, show that DA potentiates the effects of a short re-exposure protocol which is otherwise insufficient to reduce contextually conditioned fear, and also reduces spontaneous recovery and renewal of contextual and cued-fear conditioning (Haaker et al. 2013). L-DOPA also further enhances the reduction in fear produced by an effective extinction protocol, by enhancing the activity of the vmPFC (Gerlicher et al. 2018).

Considering the neurochemical changes observed during retrieval and extinction of fear memories, what neurochemical changes would be predicted to occur during the retrieval-extinction procedure? As discussed above, a typical retrieval trial (i.e. a brief CS re-exposure) might increase amygdala dopamine and serotonin levels, alongside increases in prefrontal dopamine and noradrenaline for at least an hour (Feenstra et al. 2001; Yokoyama et al. 2005) with subsequent extinction training further engaging prefrontal dopaminergic and noradrenergic signalling (Hugues et al. 2007)—i.e. a retrieval trial prior to extinction would be predicted to facilitate the dopaminergic and noradrenergic signalling that correlates with extinction learning. However, the correlative data alone do not distinguish whether increases in prefrontal dopaminergic and noradrenergic signalling alter extinction by increasing the rate of acquisition of the inhibitory extinction memory, or by enhancing its consolidation. Causal studies are more informative here, and it appears that noradrenergic and dopaminergic mechanisms underlie these two potential mechanisms respectively. Systemic injections of the α_2_ adrenergic receptor antagonist yohimbine enhance the rate of acquisition of the extinction memory (Cain et al. 2004), though subsequent studies found this effect to vary quite substantially according to individual and procedural differences (Holmes and Quirk 2010). By contrast and as discussed above, potentiation of dopaminergic signalling with L-DOPA immediately after extinction training enhances the consolidation of extinction (Haaker et al. 2013). As an increased rate of acquisition of extinction following the use of the retrieval-extinction technique has not been widely reported in the literature (Cahill et al. 2019, Monfils et al. 2009; Tedesco et al. 2014, however see also Ponnusamy et al. 2016), it would appear that the noradrenergic modulation of extinction acquisition is unlikely to account for the subsequent attenuation in fear expression associated with retrieval-extinction. Instead, the behavioural effects of L-DOPA on extinction appear to produce a more similar attenuation of fear. Increased dopaminergic tone could conceptually support either an enhanced extinction or enhanced destabilisation through increased prediction error signals; however, there is no direct evidence showing how boosting dopamine might influence destabilisation. More work is required to bolster these hypotheses, but whether extinction shortly after memory retrieval results in a different pattern of neurotransmission across these brain regions as compared to standard extinction would undoubtedly be a step towards increasing our understanding.

### Receptors required for fear memory retrieval and extinction

**Fig. 1:**
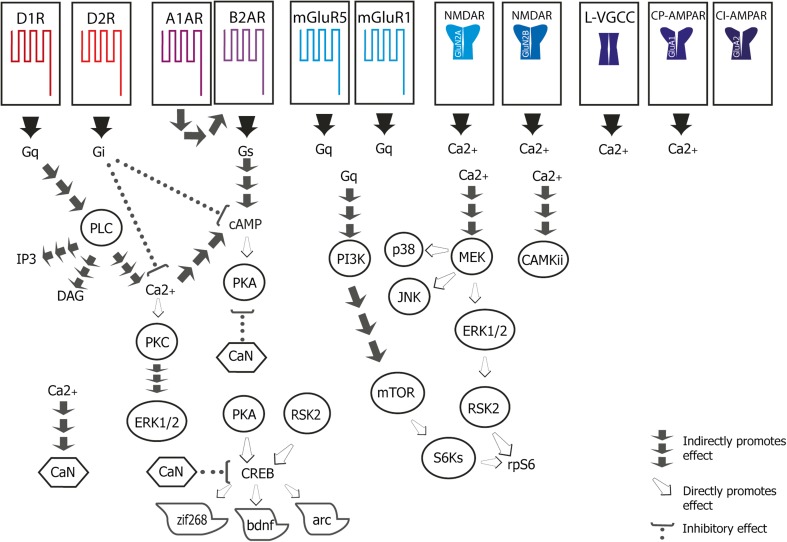
Illustration of key signalling pathways implicated in the consolidation, retrieval, extinction and/or reconsolidation of fear memories. Kinases are represented encircled. Phosphatases are represented in polygons. See text for details

The correlative studies discussed above indicate that catecholaminergic signalling has effects on both retrieval and extinction. Surprisingly, there has been little evidence of modulation of glutamatergic signalling, though as noted above, this may reflect the limitations of microdialysis in detecting rapid small releases of glutamate, rather than independence from glutamatergic signalling per se (Yokoyama et al. 2005). Due to their established role in synaptic transmission and plasticity (Traynelis et al. 2010), glutamate receptors (GlutR) have been a major focus of research for their contribution to both memory retrieval and extinction. Additionally, catecholamines can modulate synaptic plasticity through their direct and indirect influence over GlutRs via physical interactions or cell signalling (Pralong et al. 2002; Jay 2003; Terrillon and Bouvier 2004). Here, we consider the contributions of the different types of receptor to fear retrieval, reconsolidation and extinction separately for clarity, but it must be acknowledged that interactions between the receptors are likely to occur (Fig. 1).

#### Ionotropic glutamate receptors

The ionotropic glutamate receptors (iGluRs) are classed into sub-families based on their affinity for synthetic agonists: α-amino-3-hydroxy-5-methy-4-isoxazole propionate (AMPA), *N*-methyl-d-aspartate (NMDA) and kainate (Traynelis et al. 2010). We will not consider the delta family of iGluRs as they remain relatively understudied in relation to aversive conditioning (for review see Schmid and Hollmann 2008). The iGluRs share a composition of four subunits that contain four transmembrane regions, with NMDARs containing two obligatory GluN1 subunits and GluN2 (usually GluN2A or GluN2B, though less frequently GluN2C or GluN2D) and/or GluN3 subunits, and AMPARs usually existing as a ‘dimer of dimers’, containing two GluA2 subunits and two others of GluA1, GluA3 and GluA4. Ligand binding alters the conformation of the iGluR to open the ion channel. The NMDARs are permeable to monovalent cations and calcium, whereas AMPARs and Kainate receptors (KARs) are normally calcium impermeable due to expression of the GluA2 subunit (Liu and Zukin 2007).

##### AMPA receptors

AMPA receptors (AMPARs) have been extensively investigated for their role in fear memory, but distinguishing the contribution of AMPARs and KARs to mnemonic processes has been compromised by a lack of pharmacological selectivity in drugs used to target these receptors. First, considering memory retrieval and/or reactivation: the non-selective AMPAR/KAR antagonist 6-cyano-7-nitroquinoxaline-2,3-dione (CNQX) prevented retrieval of fear memory but had no effect on its destabilisation (Ben Mamou et al. 2006). Likewise, LY293558, an AMPA/GluK1 receptor antagonist, impaired fear memory retrieval but affected neither fear memory destabilisation nor restabilisation (Milton et al. 2013). Somewhat more selective compounds such as 4-[2-(phenylsulfonylamino)ethylthio]-2,6-difluoro-phenoxyacetamide (PEPA) and 1,2,3,4-tetrahydro-6-nitro-2,3-dioxo-benzo[f]quinoxaline-7-sulfonamide (NBQX) suggest more specific roles for AMPARs in fear memory processing. PEPA, a GluA3/4-preferring positive allosteric modulator of AMPARs, had no effect on the behavioural expression of fear indicative of fear memory retrieval (Zushida et al. 2007) and PEPA also had no effect on fear memory reconsolidation processes; neither destabilisation nor restabilisation (Yamada et al. 2009). Overall, these findings suggest that KAR, rather than AMPAR, may be responsible for the reduced expression of fear memory following the administration of non-specific AMPAR/KAR antagonists. This view is supported by similar findings with appetitive memories, including the observation that the non-selective AMPAR/KAR antagonist CNQX, but not the AMPAR-preferring antagonist NBQX, blocked memory expression in an amphetamine-induced conditioned place preference (CPP) task in mice, although this could also be attributed to the antagonistic actions of CNQX on the glycine site of NMDARs (Mead and Stephens 1999).

In addition to their contribution to synaptic transmission, AMPAR expression at the synapse regulates synaptic plasticity processes, with increases in synaptic AMPAR expression following long-term potentiation (LTP), and reductions following long-term depression (LTD) (Huganir and Nicoll 2013). Trafficking of AMPARs to the synaptic membrane has been associated with phosphorylation of the Ser^845^ residue of GluA1 (Ehlers 2000), and consequently phosphorylation of Ser^845^ on GluA1 has been used as a marker of synaptic plasticity. This has been used to attempt to distinguish the mechanisms underlying the retrieval-extinction effect from those underlying standard extinction, reasoning that that dephosphorylation of this site would be indicative of depotentiation of the original memory (Monfils et al. 2009). As compared to context exposure alone, a single CS presentation resulted in an almost threefold increase in pGluA1Ser^845^ within 3 min, which was still detectable an hour later (see also Holehonnur et al. 2016). If a second presentation of a CS occurred after 3 min, the pGluA1Ser^845^ signal remained high. However, if the second CS was given after 60 min, the signal returned to baseline levels. This final result suggested that the synaptic tagging of potentiated amygdala synapses (i.e. phosphorylation of GluA1) had been removed by rapid dephosphorylation induced by presentation of the second CS with a 60-min delay. The authors obtained similar results using ELISA (Monfils et al. 2009). Therefore, it could be hypothesised that the retrieval-extinction procedure causes an internalisation of AMPARs via dephosphorylation of the GluA1Ser^845^ residue. Accordingly, mutant mice with an alanine substitution for Ser^845^ did not show the reductions in spontaneous recovery and renewal normally observed after the retrieval-extinction procedure (Clem and Huganir 2010). In line with these findings, an increase in the endocytosis of GluA2 and GluA3 subunits was noted up to 4 h after retrieval. Outside of the destabilisation window (7 h), a reinsertion of GluA2-containing AMPARs was detected in the synapse (Rao-Ruiz et al. 2011).

Overall levels of synaptic AMPAR expression may not fully represent the changes associated with plasticity, which may also be correlated with changes in the relative expression of specific AMPAR subunit subtypes. As described above, AMPARs in the adult brain principally contain a complex of GluA1 and GluA2 subunits, which are impermeable to Ca^2+^ or Zn^2+^ due to the presence of a charged R residue in the channel pore (for review see Liu and Zukin 2007). The GluA3 subunit, which contains a Q rather than R residue (making it permeable to Ca^2+^ and Zn^2+^) can substitute for GluA2. Such receptors are consequently referred to as GluA2-lacking or calcium-permeable AMPARs (CP-AMPARs). Fear conditioning is correlated with transient decreases in the expression of calcium-impermeable (i.e. GluA2-containing) CI-AMPARs and increases in expression of CP-AMPARs, as shown by electrophysiological recordings and the sensitivity of transmission to a selective antagonist of CP-AMPARs, the synthetic analogue of spider venom 1-naphthylacetyl spermine (NASPM). Following cued-fear conditioning, it has been reported that expression of CP-AMPARs increases by 12–24 h after training and returns to baseline 2–7 days later (Clem and Huganir 2010; Hong et al. 2013; Park et al. 2014). Reactivation of a conditioned fear memory leads to an increase in CP-AMPAR expression in the amygdala within 5 min, returning to baseline levels within 1 h (Hong et al. 2013). Thus, transient CP-AMPAR expression correlates with the destabilisation of the fear memory. However, as neither CNQX nor LY293558 affect fear memory destabilisation, the signalling pathways engaged by this transient source of Ca^2+^ must not be essential for destabilisation to take place (Ben Mamou et al. 2006; Milton et al. 2013).

In addition to depotentiation mechanisms, various forms of LTD—a de novo decrease in synaptic efficacy—have been described as relevant to both standard extinction and retrieval-extinction. Direct comparison of standard- and retrieval-extinction has shown that levels of amygdala CP-AMPARs were reduced specifically in mice undergoing the retrieval-extinction procedure (Clem and Huganir 2010), consistent with the finding that LTD induction causes removal of CP-AMPARs, while extinction training alone did not alter AMPAR-mediated transmission (Clem and Huganir 2010). In turn, this supports the finding, noted above, that NBQX does not prevent the acquisition of extinction (Yamada et al. 2009). The authors proposed that the retrieval-extinction procedure triggered an mGluR1 and NMDAR co-activation-mediated LTD of the synapses previously potentiated by the original fear learning, i.e. that retrieval-extinction produced depotentiation of the original memory, rather than enhancement of a new extinction memory. The cell-penetrating peptide TAT-GluA2_3Y_ was designed to prevent the activity-regulated endocytosis of GluR2 containing AMPARs (Ahmadian et al. 2004). Rao-Ruiz and colleagues (Rao-Ruiz et al 2011) used this peptide to impair the effect of the retrieval-extinction technique on spontaneous recovery and therefore concluded that destabilisation had been prevented. There was no observed effect of TAT-GluA2_3Y_ on standard extinction. In contrast, GluA2_3Y_ did prevent extinction of fear memories in other studies (Dalton et al. 2008; Kim et al. 2007). Taken together, these studies point to AMPAR endocytosis as a cellular mechanism for reducing the synaptic strength of synapses necessary for altering established behaviour. Whether destabilisation or extinction is targeted by the peptide should depend on the behavioural protocol employed.

However, not all types of LTD are dependent upon CP-AMPARs. Depending on the protocol used to induce LTD—low frequency stimulation (LFS) or paired-pulse (ppLFS) procedures—the dependence on CP-AMPARs was different (Clem and Huganir 2013). ppLFS-induced LTD was blocked by NASPM, PKC inhibitors and antagonists of mGluR1—consistent with the molecular changes observed during retrieval-extinction. LFS-LTD, on the other hand, was dependent on calcineurin (PP2B—see below), which may reflect more closely the mechanisms underlying standard extinction rather than the regulation of CP-AMPAR expression associated with memory destabilisation (Clem and Huganir 2013). However, a subsequent study of thalamic to BLA synapses described that mGluR-mediated LTD neither required nor promoted removal of CP-AMPARs (Park et al. 2014). Therefore, multiple forms of LTD or depotentiation may be acting together to weaken synapses. Taken together, it might be predicted that the retrieval-extinction procedure would result in a rapid dephosphorylation of GluA1, and a decrease in the levels of CP-AMPARs, at least in the amygdala, which parallels specific forms of LTD.

##### NMDA receptors

Like AMPARs and KARs, NMDA receptors (NMDARs) are heterotetramers associated together to form a cationic channel, which in the case of the NMDAR is Ca^2+^ permeable (for review see Paoletti 2011). Unlike the AMPAR or KAR, a magnesium ion blocks the NMDAR channel pore in a voltage-dependent manner. Furthermore, NMDAR activation requires the binding of a co-agonist, with the specific co-agonist depending upon subunit composition (Kleckner et al. 1988). NMDAR subunits are grouped into three classes: GluN1, GluN2A-D and GluN3A-B (formally designated NR1 etc.). The GluN1 and GluN3 subunits can bind the co-agonists glycine (Kleckner et al. 1988) or D-serine (Mothet et al. 2000), and the GluN2 subunits contain the binding site for glutamate (Laube et al. 1997). NMDARs are ‘coincidence detectors’ and respond to the binding of glutamate only when it is accompanied by a depolarisation of the postsynaptic membrane (Mayer et al. 1984). Coincidence detection at the molecular level has placed the NMDAR as the potential ‘molecular master-switch’ to gate thresholds for plasticity and associative learning (Tsien 2000), with extensive literature refining the role of different NMDAR subunits in fear memory processes. It has been suggested that theoretically, the slower channel kinetics of GluN2B subunits provide a longer time window for coincidence detection along with larger Ca^2+^ transients relative to GluN2A subunits, and therefore the balance of GluN2B/GluN2A signalling may determine whether learning occurs (Tsien 2000; Rodrigues et al. 2001). This is supported by the findings that systemic or intra-amygdala administration of the GluN2B-preferring antagonist ifenprodil (Williams 1993) prevents learning, but not memory expression, of cued and contextual fear conditioning in a dose-dependent manner (Rodrigues et al. 2001) and by reports that ifenprodil blocks the destabilisation of fear memory without any effect on retrieval (Ben Mamou et al. 2006; Milton et al. 2013). However, systemic administration of a more selective GluN2B antagonist, Ro25-6981, did not produce any effect on fear conditioning, but did acutely impair the expression of an extinction memory without blocking the reduction in fear observed 24 h after extinction training (Dalton et al. 2012). Thus, it appears that the requirement for GluN2B-mediated signalling in learning is not straightforward.

With respect to extinction, NMDARs in the amygdala were found to be necessary for the consolidation of cued-fear extinction by intra-amygdala infusion of the non-specific antagonist 2-amino-5-phosphonovalerate (APV, Lee and Kim 1998). Similarly, systemic administration of the competitive antagonist 3-(2-carboxypiperazin-4-yl)propyl-1-phosphonic acid (CPP) affected neither the acute expression of conditioned freezing nor the acquisition of extinction memory, but did impair consolidation of the extinction memory (Santini et al. 2001; Suzuki et al. 2004). However, in addition to the ‘first wave’ of consolidation of the extinction memory, there also appears to be a ‘second wave’ of delayed memory consolidation. When animals received systemic administration of CPP following extinction training, but were only tested 48 h later, the recall of the extinction memory was unimpaired. Furthermore, if a second injection of CPP was given during the ‘rest day’, then conditioned freezing at test was impaired (Santini et al. 2001). Such results merit further investigation as many conclusions are drawn from testing only at a 24-h time point. Furthermore, the behavioural enhancement of extinction through exposure to a novel context (de Carvalho Myskiw et al. 2013) was found to be dependent on hippocampal NMDARs, as rats treated with APV after this experience did not show any facilitation of extinction (de Carvalho Myskiw et al. 2014). More selective antagonists allowed the contribution of specific NMDAR subunits to extinction to be identified. Ifenprodil infusion into the amygdala before extinction has no effect on expression of fear but prevented acquisition of the extinction memory. Thus, ‘short-term extinction’—the reduction of conditioned freezing within the session—was impaired (Sotres-Bayon et al. 2007). Similarly, Ro25-6981 prevented within-session extinction, but not the expression of fear at subsequent test (Dalton et al. 2012). These data indicate that GluN2B-NMDARs are necessary for the initial acquisition of the fear extinction memory. There was no effect of a GluN2A-preferring NMDAR antagonist, NVP-AAM077, on the acquisition of the extinction memory (Dalton et al. 2012). Considered in light of the effects of CPP on the consolidation of the extinction memory (Santini et al. 2001; Suzuki et al. 2004), this may suggest a double dissociation in the requirement for GluN2B- and GluN2A-containing NMDARs in the acquisition and consolidation of extinction memories respectively. However, this hypothesis would need to be tested with a more selective GluN2A-NMDAR antagonist, once available.

Reconsolidation of fear memory also appears to differentially recruit NMDARs depending on the stage of the memory life cycle, with the suggestion that GluN2B-NMDARs are required for memory destabilisation and GluN2A-NMDARs for restabilisation (Ben Mamou et al. 2006; Milton et al. 2013). If non-specific NMDAR antagonists are given after a reactivation session, an impairment in fear memory restabilisation is often (using systemic CPP) but not always (using intra-amygdala APV) observed (Suzuki et al. 2004; Ben Mamou et al. 2006); the variability in effect may depend on the dose and route used and preference for GluN2A- and GluN2B-NMDARs under different conditions. The GluN2B-NMDAR selective antagonist ifenprodil infused prior to reactivation prevents the amnestic effect of a subsequent infusion of the protein synthesis inhibitor anisomycin, which was interpreted as GluN2B-NMDAR antagonism preventing the destabilisation of the memory (Ben Mamou et al. 2006; Milton et al. 2013). Indeed, strong cued-fear memories that are more resistant to reconsolidation manipulations tend to correlate with lower levels of GluN2B-NMDAR expression in BLA (Wang et al. 2009; Holehonnur et al. 2016). Further support comes from recent evidence that introducing an appetitive experience after contextual fear memory reactivation only reduces fear when GluN2B-NMDAR signalling is functional, as ifenprodil prevented this updating effect (Ferrer Monti et al. 2016). When the levels of GluN2A and GluN2B expression were directly manipulated, so that the ratio of GluN2A/GluN2B was increased, a fear memory that was acquired normally was no longer able to undergo destabilisation (Holehonnur et al. 2016). Due to the lower selectivity of the antagonists, there has been less research into the requirement for GluN2A-NMDARs in memory restabilisation, but the GluN2A-preferring NMDAR antagonist NVP-AAM077 blocked the restabilisation of pavlovian fear memories (Milton et al. 2013).

The requirement for NMDAR subtypes in producing the subsequent attenuation of fear associated with retrieval-extinction has not yet been investigated. In light of the role of GluN2B in memory destabilisation, it would be predicted that if the attenuation of fear is mediated by memory updating during reconsolidation, then signalling specifically via GluN2B-NMDARs should be necessary to observe the retrieval-extinction effect. However, potentiation of NMDAR signalling alongside standard extinction procedures produces resistance to reinstatement following extinction. The partial agonist D-cycloserine (DCS) was shown to potentiate NMDAR-mediated signalling when administered after extinction training (for review, see Davis et al. 2006) and has been shown to enhance the consolidation of extinction for a cue-fear memory (Lee et al. 2006; Toth et al. 2012; Merlo et al. 2014). Rats treated with DCS after extinction training are resistant to fear reinstatement (Ledgerwood et al. 2004), and they generalise their extinction of fear responses to other fear-associated CSs (Ledgerwood et al. 2005). Recent work has highlighted that there may be limitations to DCS efficacy, as it was the rats which acquired extinction quickly (‘fast extinguishers’) that particularly benefited from the effect of DCS on relapse of fear (King et al. 2018), i.e. animals that are poor at extinction learning do not appear to benefit from DCS administration. However, despite the common reduction in fear reinstatement, there appear to be differences in the mechanisms underlying the attenuation of fear produced by DCS and retrieval-extinction; specifically, unlike DCS administration, the efficacy of the retrieval-extinction procedure does not depend on the variability in extinction learning (Auchter et al. 2017). Further work in selectively bred animals may identify predictors of extinction performance, but as of yet, no differences in any preceding exploratory behaviours could foretell how animals perform in extinction training or during retrieval-extinction (Shumake et al. 2014).

##### L-type voltage-gated calcium channels (LVGCCs)

The requirement for NMDARs in synaptic plasticity processes has been linked to their calcium permeability (Paoletti 2011), but these are not the only class of direct ion channel permeable to calcium. L-type voltage-gated calcium channels (LVGCCs) have been implicated in mnemonic processes, particularly the destabilisation of contextual fear memory (Flavell et al. 2011). As discussed above, the consolidation of an extinction memory for contextual fear can be enhanced by prior exposure to novelty; this effect was found to be dependent on activation of LVGCCs during the novelty exposure (de Carvalho Myskiw et al. 2014). However, as is the case for GluN2B-NMDARs, the effects of LVGCC activity on memory appears to depend on the dominant mnemonic process when activity is modulated. It has been noted that extinction and reconsolidation may be mutually exclusive processes (Suzuki et al. 2008), and it has previously been reported that reconsolidation and extinction can be bidirectionally modulated by giving NMDAR partial agonists and antagonists after varying degrees of re-exposure (Lee et al. 2006; Merlo et al. 2014). This also appears to be the case with antagonism of LVGCCs. Antagonism of LVGCCs with nimodipine prevented consolidation of contextual fear extinction after a long re-exposure session (Suzuki et al. 2004, 2008), but if nimodipine was given after brief contextual fear memory reactivation, it had no effect on freezing per se, but did prevent the amnestic effect of anisomycin administration. This appears to be consistent with a blockade of memory destabilisation with a short re-exposure session, though it should be noted that anisomycin was still able to prevent protein synthesis, as measured by c-Fos induction in the hippocampus, in the presence of nimodipine. LVGCCs are not required for new learning as nimodipine did not affect the acquisition of contextual fear learning (De Oliveira Alvares et al. 2013). However, although nimodipine before reactivation has no effect on the expression of contextual fear itself, when the reactivation context was made slightly dissimilar to the training context, a generalisation of fear was observed that did depend on LVGCC-mediated updating (De Oliveira Alvares et al. 2013). Consistent with a role for LVGCCs in fear memory destabilisation, nimodipine prevented the subsequent deficits in fear memory reacquisition associated with the retrieval-extinction effect if given after reactivation, but had no effect if given after the extinction phase of retrieval-extinction (Flavell et al. 2011).

In a modified protocol, a recent paper used a retrieval trial before the retrieval-extinction procedure (referred to as ‘prior retrieval’, ‘priRet’) to enhance the disruption of fear reinstatement (An et al. 2018). They demonstrated that the priRet session required LVGCC signalling, as administration of nimodipine before the session prevented the reduction in subsequent fear recovery normally observed after retrieval-extinction. To the best of our knowledge, it has not yet been shown that LVGCCs are required for destabilisation of cued-fear memories, so it is not clear whether LVGCCs are specifically required in the hippocampus for the destabilisation of contextual memories, or also in the amygdala for destabilisation of discrete cue-fear memories. The requirement for LVGCCs in the destabilisation of other memory types also requires further investigation. Both the results of GluN2B-NMDAR antagonism and LVGCC antagonism emphasise the importance of calcium-mediated signalling in the memory destabilisation process, and if the retrieval-extinction effect depends upon destabilisation of the original cue-fear memory, it would be predicted that antagonism of either system should prevent the subsequent attenuation of fear associated with retrieval-extinction.

#### Metabotropic receptors

##### Adrenergic receptors

Metabotropic receptors represent a large class of signalling proteins that are widely distributed in the central nervous system. Perhaps one of the longest studied classes of metabotropic receptors in behavioural responses to aversive experience is the noradrenergic receptors. Noradrenergic receptors exist as α and β subtypes, but despite the naming convention, the two α-noradrenergic receptors are as dissimilar to each other molecularly as they are to the β-subtype (Bylund et al. 1994). The distinction of their contributions to fear memory processing has been particularly challenging due to the potential influence of targeting one receptor class on the function of the other. Moreover, findings differ between cued and contextual fear conditioning, and the influence of noradrenaline on retrieval of those memories is also time dependent (Murchison et al. 2004). In light of this, a brief review of the potential involvement of the receptors in specific mnemonic processes is presented below, considering which components might be most relevant to the retrieval-extinction procedure.

For the α_1_-adrenoceptor, the antagonist terazosin enhanced cued-fear conditioning, as measured 24 h after treatment, when animals were drug-free and the sedative effects of the drug had dissipated (Lazzaro et al. 2010). Moreover, this enhancement of fear memory acquisition could be produced by local infusion of terazosin into the lateral amygdala prior to training, which did not result in any sedation. Infusion after training did not, however, affect fear memory consolidation (Lazzaro et al. 2010). In contrast, terazosin infusion into the VTA prior to cued-fear conditioning produced a modest reduction of conditioned freezing, but nevertheless, the animals still acquired fear conditioning (Solecki et al. 2017). The effect of terazosin on expression of fear the next day was not reported in that study, but VTA infusion of the less-selective α_1_-adrenoceptor antagonist, prazosin, did prevent freezing in response to the CS at test, without affecting measures of anxiety such as exploration or 22 kHz ultrasonic vocalisations (Solecki et al. 2017). Prazosin also influences contextual fear extinction (Bernardi and Lattal 2010; Do-Monte et al. 2010a). Post-session systemic administration of a high dose of prazosin was found to retard the acquisition of contextual fear extinction over a number of sessions and days (Bernardi and Lattal 2010), but this effect was not seen at lower doses (Do-Monte et al. 2010a). If prazosin was administered before each extinction session, there was a deficit in the acquisition of extinction, and this was also seen if local administration to the mPFC was performed (Do-Monte et al. 2010a). These data indicate that systemic antagonism of α_1_-adrenoceptors promotes fear responses at acquisition and extinction. Surprisingly, however, there is some clinical evidence in humans that supports prazosin as a treatment for PTSD (Taylor et al. 2008). As systemic or local injection of prazosin directly into the prelimbic cortex after a memory reactivation session disrupted the reconsolidation of an odour-cued-fear memory (Do Monte et al. 2013), it might be that prazosin acts to reduce fear memory strength through reconsolidation-based mechanisms rather than extinction.

With respect to α_2_-adrenoceptors, the actions of antagonists must be interpreted with caution, as antagonsim of α_2_-adrenoceptors in presynaptic terminals can promote an increase in noradrenaline release. The antagonist yohimbine, which is thought to act by decreasing the inhibitory influence of the α_2_-adrenoceptor on noradrenaline release, enhanced contextual fear memory consolidation. Yohimbine caused some generalisation of fear to an unpaired context, but this effect was prevented by pre-treatment with the β_2_-adrenergic receptor antagonist propranolol, indicating that yohimbine was acting through increasing noradrenergic signalling (Gazarini et al. 2013). If yohimbine treatment was given after a reactivation trial rather than post-acquisition, freezing was enhanced at test 24 h later, indicating that yohimbine also enhances memory reconsolidation. The increase in freezing induced by post-reactivation administration of yohimbine could be prevented with pre-treatment of either prazosin or propranolol, meaning that α_1_- and β_2_-adrenoreceptors must both contribute to the actions on reconsolidation.

Conversely, yohimbine has been shown to reduce fear by enhancing the acquisition of both cued and contextual fear extinction (Cain et al. 2004). Yohimbine acted on within-session extinction, and not its consolidation, as no effect was seen if the drug was administered after extinction training. The effects of yohimbine have been discussed in terms of its actions of the contextual regulation of extinction learning, as it appears to enhance extinction only when there is a context shift (Morris and Bouton 2007; Mueller and Cahill 2010). However, the clinical utility of yohimbine is somewhat limited due to its anxiogenic nature. Despite this practical issue in exposure therapy sessions (Mueller and Cahill 2010), yohimbine is still being tested as an adjunct to exposure therapy (Meyerbröker et al. 2012). A number of questions remain about the specificity of yohimbine to α_2_-adrenoceptors (Holmes and Quirk 2010). Nonetheless, such findings may still inform the reduction of fear in the laboratory setting. As discussed above, it seems that only manipulations that influence extinction memory consolidation, not the rate of extinction acquisition, influence the extent of fear memory recovery. Thus, based on the effects of yohimbine in enhancing the rate of acquisition but not consolidation of extinction, then it would be predicted that yohimbine would not reduce fear relapse (i.e. reinstatement, spontaneous recovery, renewal of reacquisition). To date, this has not been investigated.

Agonists of α_2_-adrenoceptors have also showed promise to reduce fear, as clonidine was reported to impair both the consolidation and reconsolidation of contextual fear memory (Gazarini et al. 2013). However, it is the β-adrenergic receptors that have received the most attention with respect to fear memory reconsolidation. Propranolol was the first receptor antagonist shown to disrupt the reconsolidation of pavlovian fear memories, though it had no effect on fear memory consolidation (Dębiec and LeDoux 2004). However, subsequent work has shown that in a multiple fear-conditioning session protocol, propranolol does prevent the enhancing effect of increased noradrenaline transmission on fear expression, and reduces fear memory when tested the following day (Díaz-Mataix et al. 2017). In experiments where DCS was used to facilitate memory reconsolidation, propranolol after the reactivation blocked this effect (Yamada et al. 2009), suggesting that β-adrenergic receptors also contribute to the reconsolidation of contextual fear memory. Agonism of β-adrenoceptors by isoproterenol in the lateral amygdala after reactivation enhanced freezing measured at test 2 days later (Dębiec et al. 2011), reminiscent of the effects of yohimbine, described above (Cain et al. 2004). The increase in freezing induced by isoproterenol could be reversed by infusion of propranolol at a second reactivation trial, suggesting that β-adrenergic receptors can bidirectionally modulate fear memory strength following reactivation, consistent with findings in appetitive memory reconsolidation (Schramm et al. 2016).

There has been discussion in the literature over whether the effects of propranolol on fear memory expression are mediated via a blockade of reconsolidation or a facilitation of extinction mechanisms (Giustino et al. 2016). Certainly, β-adrenergic receptors have been shown to modulate the extinction of fear, but the effects are complicated and appear related to the type of protocol used to induce extinction. Acute treatment with propranolol before massed extinction training had no effect on cued-fear extinction acquisition or expression in mice (Cain et al. 2004), but a later study showed that propranolol administered before extinction training led to an acute reduction in freezing, but with no effect on the rate of extinction learning or consolidation of the extinction memory. Furthermore, both propranolol-treated and control groups showed equivalent levels of fear reinstatement to a reminder shock (Rodriguez-Romaguera et al. 2009). Local administration of propranolol to the infralimbic cortex had no effect on the expression of freezing but did prevent consolidation of cued-fear extinction (Mueller et al. 2008). For contextual fear extinction, multiple systemic administrations of propranolol had a dose-dependent effect on the acquisition of extinction (Do-Monte et al. 2010b). Thus, there could be a significant region-specific and protocol- specific contribution of β-adrenergic receptors to extinction that is not sufficiently targeted by acute systemic application of the drug.

Heightened arousal has been proposed to enhance extinction, and this appears to be mediated via a β-adrenergic mechanism (Luo et al. 2015). The consolidation of contextual fear extinction memory was enhanced by placing animals in an open-field novel environment 1 h, but not 6 h, before extinction training. This novelty-enhanced extinction lasted up to a week after extinction training and was resistant to reinstatement. The same effect could also be seen by giving rats the novel environment experience 1 h after extinction training. Propranolol infusion directly into the CA1 region of the hippocampus prevented the novel context enhancement of extinction if the infusion was coupled to the novelty exposure either pre- or post-extinction training. However, administration of propranolol before the extinction session (whether preceded or followed by novelty) did not affect the enhancement of extinction, suggesting that the signalling engaged by novel context exposure must require hippocampal β-adrenergic receptors, but during extinction, these receptors are no longer required (Liu et al. 2015).

##### Dopamine receptors (DARs)

As evidence accumulates for a role of dopamine as a learning signal in aversive memory (Ilango et al. 2012), it is unsurprising that its receptors have been heavily investigated in the regulation of fear memories. Dopamine receptors are classed into two families, namely D_1_-like and D_2_-like receptors, based on the coupling to different classes of G protein and their regulation of adenylyl cyclase activity (for review see Beaulieu and Gainetdinov 2011). The acute effects of dopamine receptor antagonism on conditioned freezing (Guarraci et al. 1999) complicate analysis of the requirement for dopamine in fear memory reconsolidation and extinction. However, it has been observed that SCH-23390 infusions into the BLA prevent within-session acquisition, but not consolidation, of cued-fear extinction, whereas infusions into the infralimbic cortex (IL) only impair the consolidation of extinction (Hikind and Maroun 2008). If the D_1/5_-receptor agonist SKF81297 was administered systemically after extinction, it enhanced consolidation of extinction of cued fear and there was a trend for reduced renewal in the drug-treated mice (Abraham et al. 2016). This finding was replicated using another unbiased D_1_R agonist (SKF83822) but not a cAMP-biased agonist (SKF83959), which might suggest that β-arrestin-mediated signalling accounts for the enhancement of extinction consolidation through D_1_-like receptors.

Infusion of the D_2_-receptor antagonist raclopride into the IL also prevented the consolidation of extinction, without affecting acquisition of the extinction memory, an example of where targeting D_1_Rs and D_2_Rs pharmacologically produced the same behavioural effect (Mueller et al. 2010). Interestingly, D_2_R activation in structures beyond the mPFC and amygdala is also implicated in controlling extinction, as D_2_R antagonism with haloperidol in the nucleus accumbens (NAc) prevents both the acquisition and consolidation of cued-fear extinction (Holtzman-Assif et al. 2010). Systemic use of the D_2_R antagonist sulpiride before extinction training showed an enhancement of extinction the next day at test, which was attributed to effects on consolidation (Ponnusamy et al. 2005). Therefore, region-specific contributions must again be carefully considered before targeting a subset of receptors.

The contribution of dopamine receptors to reconsolidation has been less extensively studied than their contribution to extinction. Systemic treatment with SCH-23390 before or after reactivation of a contextual fear memory did not block restabilisation of that memory (Heath et al. 2015) but whether the D_1_Rs could be contributing to the destabilisation of fear memory—as they do for appetitive memories (Merlo et al. 2015)—was not assessed. Very few studies have tried to address the exact role of DA receptor subtypes in reconsolidation, and it remains understudied, especially in light of the discussion of a potential role of DA as an error prediction signal also in aversive situations (Brischoux et al. 2009).

##### Metabotropic glutamate receptors (mGluRs)

The group 1 metabotropic glutamate receptors (mGluR), mGluR1 and mGluR5, are positively coupled to phospholipase C (PLC, see below for discussion of signalling cascades) and are thought to act as modulators of synaptic plasticity involved in fear conditioning and extinction (Johansen et al. 2011). The mGluR1 antagonist CPCCOEt, administered before extinction training, impaired the acquisition of extinction in a dose-dependent manner (Kim et al. 2007). The effect of CPCCOEt was also dependent on the time since training, as a dose that disrupted extinction conducted 48 h after conditioning had no effect 2 h after conditioning, despite levels of freezing being quantitatively similar. Antagonism of mGluR5, which belongs to the same family as mGluR1, either systemically or in the IL prevented the consolidation of extinction memory (Fontanez-Nuin et al. 2011). Following this finding, it was hypothesised that potentiation of mGluR5 signalling might be able to enhance fear extinction, but a positive allosteric modulator of mGluR5, ADX47273, administered before contextual fear extinction did not affect the acquisition of extinction, even at a relatively high dose (Xu et al. 2013). Similar findings were reported for cued-fear extinction. Furthermore, a study using MPEP before extinction training noted no effect on cued-fear extinction acquisition or consolidation (Toth et al. 2012). Therefore, mGluR5 activation might not be a viable strategy to enhance extinction.

The roles of mGluR1 and mGluR5 in the retrieval-extinction procedure seem more complex. It has been argued that mGluR1 activation is required for the success of the retrieval-extinction procedure (Clem and Huganir 2010). Administration of the mGluR1 antagonist 1-aminoindan-1,5-dicarboxylic acid (AIDA) before the retrieval trial resulted in a lesser attenuation of spontaneous recovery and renewal associated with retrieval-extinction. The mechanism was proposed to involve an mGluR1-dependent removal of CP-AMPARs, but further studies have demonstrated that mGluR1-mediated depotentiation was not differentially sensitive to NASPM, indicating that CP-AMPAR removal does not always occur during amygdala depotentiation (Park et al. 2014). Modulation of mGluR5-mediated signalling with the positive allosteric modulator ADX47273 prior to retrieval enhanced the attenuation of spontaneous recovery and renewal associated with retrieval-extinction, under conditions in which control animals did not show any fear attenuation. However, there were no differences between controls and ADX47273-treated animals at test 27 days later, indicating that the memory had not been erased or permanently weakened (Xu et al. 2013). The mechanism of this action is not clear, and local administration studies into the BLA and IL would be useful to delineate the site of action of mGluR5-mediated signalling.

The Group II mGluRs, mGluR2 and mGluR3, are negatively coupled to adenylyl cyclase production. Specific drugs to target each receptor class have been relatively challenging to develop (Nielsen et al. 2011), and many act at both mGluR2s and mGluR3s. Activation of mGluR2/3s appears to be necessary for fear memory extinction, as the non-selective mGluR2/3 antagonist LY341495 infused directly in the BLA impaired the consolidation of extinction (Kim et al. 2015). However, the mGluR2/3 agonist LY317206 did not significantly affect fear expression and had no effect on fear destabilisation nor restabilisation (Milton et al. 2013), suggesting that the contribution of mGluR2/3s may be specific to extinction.

The study of group III mGluRs has also been limited due to the shortage of selective pharmacological compounds. mGluR4 has been targeted using the agonist LSP1-2111, which has mild effects on the acquisition of fear extinction (Davis et al. 2013). However, the positive allosteric modulator of mGluR4, ADX88178, affected neither the acquisition nor the consolidation of extinction (Kalinichev et al. 2014). The allosteric agonist of mGluR7, AMN082 (N,N0-dibenzyhydryl-ethane-1,2-diamine dihydrochloride), can bidirectionally modulate extinction depending on the protocol used to induce extinction learning. AMN082 before massed extinction training acutely increased the expression of freezing and prevented within-session extinction, without affecting the consolidation of extinction memory (Toth et al. 2012). However, when administered after a brief, suboptimal extinction training protocol that did not successfully extinguish responding in controls, AMN082 facilitated the consolidation of extinction (Toth et al. 2012). Whether this enhanced extinction produced resistance to any forms of relapse was not examined. These results may be attributable to regional effects of the drug. When AMN082 was administered locally to the BLA, though not the mPFC, extinction of contextual fear was enhanced within session but was not different to controls at subsequent test (Dobi et al. 2013; Morawska and Fendt 2012). Similarly, BLA administration of (S)-3,4-DCPG, a mGluR8 agonist, enhanced within-session extinction (Dobi et al. 2013). These data support the differential contribution of the BLA and mPFC circuits to the acquisition and consolidation of extinction learning. Whether this class of mGluR also contributes to memory reconsolidation remains to be established.

## Intracellular signalling pathways required for (retrieval-) extinction

The activation of metabotropic receptors leads to the initiation of a range of intracellular signalling pathways that first engage second messengers, followed by effector proteins or third messengers (Fig. 1). Under certain conditions, this activation can ultimately lead to alterations in the activation of transcription factors and protein synthesis. The intracellular signalling pathways recruited by specific receptors can be determined in correlative, post-mortem studies, and in some cases causally with pharmacological agents. Despite the caveat that current post-mortem techniques provide a limited snapshot of the signalling engaged by a mnemonic process, they can provide useful markers to identify which pathways may be recruited and required for the maintenance and modification of specific memories.

### Second messenger pathways

#### cAMP/PKA

Adenylyl cyclase (AC) is a widely studied hub in signalling related to learning and memory. It regulates plasticity processes through the activation of adenosine 3′,5′-cyclic monophosphate (cAMP)-dependent protein kinase A (PKA). cAMP-mediated activation of PKA can be inhibited by Rp-cAMPS, which therefore prevents PKA activity. Another approach is to prevent PKA from anchoring correctly to its scaffolds using interfering peptides such as St-superAKAP-IS (Nijholt et al. 2008). St-superAKAP-IS was shown to accelerate contextual fear extinction if given after each trial in a spaced extinction protocol over 7 days (Nijholt et al. 2008). However, Rp-cAMPS infusion to the hippocampus did not produce an impairment in extinction learning using a similar spaced protocol (Tronson et al. 2008) and consistently, the activation of PKA in the BLA with N6-benzoyladenosine-3′,5′-cyclic monophosphate (6-BNZ-cAMP) after each extinction session had no effect on cued-fear extinction (Tronson et al. 2006). There is a greater evidence base implicating the activation of PKA in the restabilisation of memory. Artificial activation of PKA with 6-BNZ-cAMP after reactivation enhanced fear memory expression on subsequent days, even in absence of a shock, as if the memory had become ‘stronger’ following reactivation. When PKA activation was inhibited after a reactivation trial, memory reconsolidation was impaired (Tronson et al. 2006). The requirement for PKA in the retrieval-extinction procedure has not yet been examined. Based on these studies, PKA activation may be a shared substrate of consolidation and reconsolidation of fear memories, but not required for their extinction.

How might the activation of PKA be linked to the neurotransmitter receptors discussed above? This may vary across structures, as it is of note that the origins of the PKA activation in the amygdala may differ from other brain areas. Although signalling at D_1_Rs leads to activation of the cAMP/PKA pathway in most brain regions, unusually, D_1_Rs do not appear to couple to this pathway in amygdala nuclei (Leonard et al. 2003). Thus, it is more likely that the major source of cAMP/PKA signalling originates from the noradrenergic receptors. Noradrenaline could activate both postsynaptic α_1_ARs and β_2_ARs, but it has been shown, at least in striatal preparations, that agonists of the α_1_AR do little other than potentiate cAMP activation by β_2_AR (Leblanc and Ciaranello 1984). It is possible that cAMP in the amygdala may be regulated by synergistic activation of both adrenoceptors. In short, a number of parallels can be seen between pharmacological manipulation of the β_2_AR and PKA, as reducing activation of both prevents reconsolidation while increasing activation enhances it (Dębiec and LeDoux 2004; Tronson et al. 2006; Dębiec et al. 2011). However, propranolol does not block consolidation of cued fear (Dębiec and LeDoux 2004) whereas PKA inhibition does (Schafe and LeDoux 2000). This may suggest that different sources are recruited for PKA activation in memory consolidation and reconsolidation. It is therefore difficult to predict how modulation of PKA would influence fear memory relapse following retrieval-extinction, especially as blockade could potentially result in the same behavioural consequence of reduced fear: i.e. enhanced extinction (Nijholt et al. 2008) but also a disruption of fear memory restabilisation (Tronson and Taylor 2007).

#### PLC/PKC

Activation of phospholipase C (PLC) leads to increases in inositol trisphosphate (IP_3_), and can also lead to downstream activation of isoforms of protein kinase C (PKC). As noted above, amygdala D_1_Rs do not signal through PKA pathways (Leonard et al. 2003), but rather appear to be coupled to G_q_ proteins, which in turn act via PLC (Jin et al. 2001). This could account for the recent finding that a cAMP-biased D_1/5_R agonist did not affect contextual fear extinction whereas a broader dopamine receptor agonist—which could also act via PLC—did (Abraham et al. 2016). However, as the treatments in this study were systemic, it is hard to determine whether biased signalling in one region is responsible for the behavioural effect. Certainly, the activation of PKC seems to oppose fear memory extinction, as PKC inhibitors enhanced the extinction of contextual fear and reduced subsequent fear reinstatement (Tronson et al. 2008).

In addition to enhancing the extinction of fear memories, the inhibition of PKC also impairs the reconsolidation of contextual fear memories, while an activator increased memory strength (Girardi et al. 2016). These findings are reminiscent of those regarding PKA described above and might suggest common targets. Indeed, when both PKA and PKC were targeted by the inhibitor 1-(5%-isoquinolinesulfonyl)-2-methylpiperazine (H7), consolidation of both cued and contextual fear was prevented (Goosens et al. 2000) but it remains to be tested whether this is also true for fear memory reconsolidation and/or extinction.

PLC may provide a link between mGluR1/5s and CP-AMPARs, both of which have been shown to be recruited by the retrieval-extinction procedure (Clem and Huganir 2010). As discussed above, CP-AMPARs are thought to contain the GluA1 subunit. Animals that underwent the retrieval-extinction procedure were less sensitive to the GluA2-selective AMPAR antagonist NASPM than those that underwent standard extinction training, suggesting that the retrieval-extinction procedure may lead to removal of CP-AMPARs from the synaptic membrane. To the best of our knowledge, no direct link between mGluR1 and pSer^845^GluA1 has been made. The Ser^845^ site is phosphorylated by PKA, but as group I mGluRs do not couple to inhibition of cAMP/PKA, the effects would need to be indirect. The mGluR1/5 family may also signal through PLC pathways (Johansen et al. 2011). mGluR1-mediated LTD has been described as generated by postsynaptic induction and expression (Bellone et al. 2008). At least in the VTA, this LTD requires mTOR and de novo synthesis of GluA2, switching them for the CP-AMPAR (GluA2-lacking). One could predict that the opposing process could occur, instead of or in parallel to GluA1 dephosphorylation and removal; that activation of PKC at retrieval could lead to phosphorylation of pSer^880^ GluA2 and boost the insertion of the CP-AMPARs.

### Third messenger pathways

Cytoplasmic signals ultimately regulate gene expression for long-term changes in behaviour. To alter the control of nuclear events, a network of kinases and phosphates regulate transcription factors, and ultimately protein synthesis.

#### The RSK2-rpS6 pathway

The phosphorylation of S6 ribosomal protein (rpS6) was proposed as a marker of reconsolidation, as in the IL, PL and lateral amygdala, phosphorylation of Ser^235/236^ on rpS6 was increased by the retrieval-extinction procedure, but not by retrieval alone (Tedesco et al. 2014). The phosphorylation of rpS6 was initially investigated because of its potential implication in the control of translation downstream of mTOR signalling (Tedesco et al. 2014). However, definitive data implicating the mTOR pathway are lacking: phosphorylation of these sites is not blocked by rapamycin (Roux et al. 2007), and instead, it may be mediated by the ribosomal protein S6 kinases (RSKs) (Pende et al. 2004). Notably, RSK2 knockout mice have been extensively characterised for their learning and memory deficits, with knockouts showing overexpression of *gria2* (which encodes GluA2 subunit of the AMPAR) and a mild impairment in the reconsolidation of contextual fear memory (Morice et al. 2013). This leads to the prediction that inhibition of RSK2 may prevent the subsequent attenuation of fear produced by the retrieval-extinction procedure, but to date, this has not been empirically tested.

#### The MAPK pathways

The requirement of extracellular signal-regulated kinase (ERK; also mitogen-activated protein kinase, MAPK) for memory per se has been extensively reviewed elsewhere (Thomas and Huganir 2004; Cestari et al. 2013), so here, we consider the specific contributions of ERK to reconsolidation and extinction, and how it may be required during the retrieval-extinction procedure. ERK is necessary for the reconsolidation (Duvarci et al. 2005) and extinction (Merlo et al. 2018) of discrete pavlovian cue-fear memories. However, for contextual fear, inhibition of MEK (MAPKK) with U0126 prevents consolidation of the extinction memory (Fischer et al. 2007), but not reconsolidation of the contextual fear memory (Lee and Hynds 2013). We speculate that this represents the differential dependence of cued or contextual fear memory on the amygdala and hippocampus respectively. In light of this, and the finding that the phosphorylation of ERK increases both during reconsolidation and extinction (Merlo et al. 2014), ERK would likely prove unsuitable for disentangling the plasticity mechanisms underlying the retrieval-extinction procedure.

The other major members of the MAPK family, JNK and p38, are relatively less studied for their contribution to learning and memory, but evidence of their necessity for mnemonic processes is building. It was initially suggested that the amnestic effects of anisomycin given at reactivation could be due to activation of JNK and p38 rather than protein synthesis inhibition (Duvarci et al. 2005). Convergent evidence from the use of other protein synthesis inhibitors, such as cycloheximide—which at least does not activate p38 (Moult et al. 2008)—argues against this idea (Duvarci et al. 2005). However, it still has not been directly investigated whether p38 or JNK activation does influence reconsolidation. In contextual fear memory consolidation, there was a short-lived but significant increase of JNK phosphorylation after 1 h (Sherrin et al. 2010). If JNK activity in the dorsal hippocampus was prevented at this time point, learning could be enhanced, leading to the suggestion that JNK may act as a break on memory, opposing the ERK signalling pathway (Sherrin et al. 2010). Moreover, the negative effects of stress on consolidation could be mimicked by anisomycin treatment after training, but not if it was combined with an inhibitor of JNK. This finding bolsters the idea that some actions of anisomycin on memory consolidation may be independent of its effects on protein synthesis and instead rely on control of JNK activity. JNK1 has also been investigated for its potential contribution to strengthening of memory (Leach et al. 2015). *jnk1* knockout mice can acquire both cued and contextual fear, but have a specific deficit in learning from multiple trials in contextual fear. The authors propose that JNK1 may not be necessary for learning but is needed for enhanced learning from extra trials—perhaps suggesting an involvement in reconsolidation (Lee 2008).

At the molecular level, JNK1 appears to be involved in the trafficking of the GluA2 subunit of the AMPAR (Thomas et al. 2008). Under basal conditions, JNK1 was not required for basal recycling of the subunits, but in response to glutamatergic activation, the GluA2L (Long isoform) subunit becomes internalised via dephosphorylation of the Thr^912^ site, which is a substrate of constitutively active JNK1. Due to the proposed role of GluA2 insertion (i.e. CI-AMPARs) in the stabilisation of fear memories (Clem and Huganir 2010), JNK1 may contribute to this process in the amygdala.

The other major member of the MAPK family of kinases is p38, which is thought to act in a manner closer to JNK rather than ERK. In the hippocampus, mGluR5-dependent LTD is blocked by inhibition of p38 (Bolshakov et al. 2000; Moult et al. 2008). At the behavioural level, few functional connections to conditioned fear memory mechanisms have been established. However, contextual fear memory consolidation can be enhanced by treatment with heat-shock protein 70 (HSP701), which acts through blocking p38 and JNK activity, which the authors suggest may lead to an upregulation of ERK activity (Porto et al. 2018).

#### Ca2+/CaMKII

The calcium-calmodulin-dependent protein kinase II (CaMKII) has been widely studied for its contribution to cued and contextual fear memory consolidation (Rodrigues et al. 2004) and more recently, CaMKII has been attributed a role in memory strengthening and reconsolidation (de Carvalho Myskiw et al. 2014; Jarome et al. 2016; Vigil and Giese 2018). CaMKII is found in close proximity to GluN2B-NMDARs in the amygdala (Rodrigues et al. 2004). CaMKII has been proposed to contribute to specifically destabilisation of contextual fear through regulation of proteasome activation (Jarome et al. 2016). In support of this, the effect of anisomycin on restabilisation is prevented if CaMKII is inhibited in the amygdala (Jarome et al. 2016). In terms of cued or contextual fear memory extinction, the role of CaMKII has not been very directly ascertained, but facilitation of extinction by novelty was prevented by the substrate-based inhibitor AID (de Carvalho Myskiw et al. 2014). It is also noteworthy that for appetitive cue-drug memories, inhibition of CaMKII not only disrupts reconsolidation, but also appears to facilitate extinction (Rich et al. 2016). If the retrieval-extinction effect depends upon memory updating mechanisms, it might be predicted that retrieval engages CaMKII-mediated destabilisation. How CaMKII is recruited for cued or contextual fear extinction is unclear.

#### Calcineurin (PP2B)

Phosphatase activity is central for immediate signalling responses and to control signal duration (Nguyen et al. 2013). The serine/threonine protein phosphatase calcineurin (calcium-dependent phosphatase 2b) is likely recruited during the retrieval-extinction procedure, due to its interactions with other molecules implicated in both reconsolidation and extinction. However, the requirement for calcineurin in extinction learning appears to differ between the amygdala and hippocampus, and cued and contextual fear respectively.

Calcineurin is found both pre- and post-synaptically and can be anchored to PSD complexes along with receptors, via scaffolds such as AKAP75/79/150 (Mansuy 2003). Inhibitors of calcineurin decrease desensitisation of GluN2A-containing NMDARs, by preventing calcineurin from dephosphorylating Ser^900^ and/or Ser^929^ of the c-terminal tail of GluN2A (Krupp et al. 2002). Furthermore, calcineurin may also modulate plasticity at the level of the nucleus, as early in vitro evidence from primary hippocampal neurons showed that calcineurin can dephosphorylate CREB (Bito et al. 1996). Together, this evidence suggests that calcineurin may act as a brake on potentiation and synaptic strengthening, consistent with the finding that calcineurin is necessary for depotentiation in amygdala slices (Lin et al. 2003).

Behaviourally, the effects of calcineurin appear to differ between the amygdala and hippocampus. In hippocampal-dependent contextual fear conditioning, knockdown of calcineurin with antisense ODNs (Ikegami and Inokuchi 2000) or inhibitors such as FK-506 (de la Fuente et al. 2014) facilitates the consolidation of the fear memory. However, the indirect inhibitor of calcineurin, cyclosporine (CsA), did not affect fear conditioning (Almeida-Correâ et al. 2015). With respect to extinction learning, both intracerebroventricular CsA and FK-506 prevented within-session extinction of contextual fear, without affecting its consolidation (Almeida-Correâ et al. 2015). Similar findings were also reported for within-session extinction of cued fear (Almeida-Correâ et al. 2015). However, inhibition of calcineurin with FK-506 during the reactivation of a contextual fear memory *strengthens* the original fear memory, leading to speculation that calcineurin activity impairs reconsolidation while facilitating extinction (de la Fuente et al. 2014). However, this bidirectional modulation of reconsolidation and extinction appears to be specific to the hippocampus. In the amygdala, the activation of calcineurin was not significantly increased by cued-fear memory reactivation (Merlo et al. 2014) and in contrast to hippocampal manipulations, in the amygdala, knockdown of calcineurin after reactivation had no effect on memory reconsolidation, though it did prevent the consolidation of extinction (Merlo et al. 2014).

Therefore, at least in the amygdala, calcineurin provides a useful marker of extinction, as it appears selectively activated during extinction learning and there is a negative correlation between calcineurin expression and conditioned freezing (Merlo et al. 2014). The expression of calcineurin could therefore be used to determine whether retrieval-extinction engages a reconsolidation-updating mechanism, or facilitates extinction. If retrieval-extinction enhances consolidation of the extinction memory, then it would be predicted that the retrieval-extinction procedure would lead to enhanced expression of calcineurin as compared to standard extinction training.

## Transcription factors and immediate early genes required for fear memory reconsolidation and extinction

The pathways described so far converge upon the control of transcriptional events which permit long-term changes in the cell. The regulation of transcription factors permits a fine control of nuclear events in functionally appropriate time windows. Often, the products of these rapidly induced events are used as ‘markers’ of neuronal activation, so-called immediate-early genes (IEGs); however, understanding of the functional consequences of this activation remains limited. Despite this, resolution of the roles of transcription factors and IEGs in learning and memory has steadily grown in recent years.

Activation of the PKA pathway, among other stimuli, can lead to phosphorylation of CREB. CREB can integrate signals from cAMP/PKA and Ca^2+^-triggered cascades and becomes activated by phosphorylation at Ser^133^, leading to induction of *c-fos* and its product Fos (Sheng et al. 1990). CREB targets the promoter of genes which contain a Ca^2+^ response element (CRE) sequence. CRE-mediated gene transcription occurs throughout the brain, but there are regional differences in the contribution of CRE-mediated transcription to extinction and reconsolidation. Forebrain knockdown of CREB expression in inducible repressor mice (CREB^IR^ mice) affected neither the expression of fear nor acquisition of contextual fear extinction, but impaired consolidation of the extinction memory (Mamiya et al. 2009). CREB phosphorylation at Ser^133^ was induced in both the prelimbic (PL) and infralimbic (IL) cortex after extinction training but not after memory reactivation in these structures. In contrast, reactivation induced a rapid increase in pCREB in CA1 and CA3, but not the dentate gyrus, of the hippocampus. However, in the basolateral, lateral and central amygdala, pCREB increased rapidly after reactivation and after extinction training. As pCREB only increases in the mPFC following extinction, and anisomycin infusion into the mPFC did not disrupt reconsolidation (Mamiya et al. 2009), it can be predicted that pCREB expression could be used to determine whether the retrieval-extinction procedure depends upon reconsolidation-based mechanisms; if an updating of the original memory, then extinction learning should not be engaged and no changes in pCREB expression would be observed in the mPFC.

A more recently explored IEG downstream of ERK/CREB pathways for cued-fear conditioning in the amygdala is activity-regulated cytoskeletal-associated protein (Arc, Ploski et al. 2008), which is of particular interest due to its known interactions with AMPARs (Lonergan et al. 2010). In a similar fashion to the pattern of pCREB induction, the IEG *arc* was induced in the amygdala nuclei and hippocampus by reactivation, and in the IL, PL and amygdala by extinction training (Mamiya et al. 2009). Arc expression in the amygdala is required for the extinction of contextual fear conditioning (Onoue et al. 2014; Germeroth et al. 2017), and Arc has been associated with retrieval-extinction. There is an increase in *arc* mRNA in the IL and PL after a 10 CS (standard) extinction protocol versus a 1 + 4 CS (retrieval-extinction) protocol (Lee et al. 2016). However, it was also observed that Arc expression may not be linked to associative learning, but rather to stimulus presentation. Moreover, as these are correlative rather than causal data, any interpretation of Arc being a marker of the recruitment of reconsolidation-update mechanisms during the retrieval-extinction procedure remains speculative.

Another product downstream of the CRE promoter is *zif268*, although this also contains a serum response element promotor (SRE) and so can be regulated by other pathways. A series of studies at the turn of the twenty-first century laid out a system of transcription factors and IEGs recruited by the reactivation of contextual and cued-fear memory. For contextual fear memory, Zif268 expression increased in the lateral amygdala (Hall et al. 2000) and knockdown of *zif268* dose-dependently blocked the consolidation of a contextual fear memory (Malkani et al. 2004). A ‘gene dose-dependent’ effect of *zif268* was also observed, as homozygous knockout mice displayed a deficit in consolidation of contextual fear conditioning, while heterozygote mice showed normal consolidation but a deficit in restabilisation following reactivation (Besnard et al. 2013). When the contextual fear memory was reactivated, Zif268 expression was increased in both the hippocampus and amygdala for contextual fear conditioning (Hall et al. 2001a). However, cued-fear memory reactivation only increased Zif268 levels in the amygdala and not in the hippocampus (Hall et al. 2001a). Interestingly the induction of Zif268 in the hippocampus for contextual fear was time-limited, as reactivation of a 28-day-old memory did not alter Zif268 expression despite equivalent levels of conditioned freezing (Hall et al. 2001a). This suggests that the activation of Zif268 in the hippocampus by retrieval was not due to expression of the behaviour, but rather related to plasticity events. For cued-fear memory, CREB was also noted to be activated by reactivation of a cued-fear memory in the central and lateral amygdala but not hippocampus (Hall et al. 2001b). This is consistent with the subsequent demonstration of a double dissociation for the requirement of BDNF and Zif268 in the hippocampus for contextual fear memory consolidation and reconsolidation respectively (Lee et al. 2004). However, this double dissociation may be specific to the dorsal hippocampus, as in the amygdala Zif268 is required for both the consolidation and reconsolidation of fear memories (Maddox et al. 2010).

Zif268 expression is also observed to change in the mPFC following memory reactivation. Inactivation of the PL with the GABA_A_R agonist muscimol during contextual fear memory reactivation disrupted the restabilisation of both 7- and 21-day-old memories (Stern et al. 2014). Muscimol infused into the IL had no effect on memory persistence. Furthermore, consistent with Zif268 having a role in updating memories via reconsolidation, rats that had undergone reactivation showed greater numbers of Zif268-positive cells in the PL but not IL (Stern et al. 2014). However, these data differed from those collected following retrieval-extinction (Tedesco et al. 2014). Whilst retrieval-extinction led to an increase in amygdala Zif268 and no change in the hippocampus, replicating earlier findings (Hall et al. 2001a), there was also an increase in Zif268 in both the IL and PL, which is apparently discrepant with the findings of Stern et al. (2014). Extinction training alone had no significant effect on Zif268 expression in any region examined (Tedesco et al. 2014). The functional implications of increased Zif268 expression, however, remain unclear.

## Conclusion: does the retrieval-extinction effect reflect rewriting or repression?

The attenuation of fear memory produced by the retrieval-extinction procedure has generated much interest in the decade since its discovery. However, it remains to be established whether the mechanisms underlying the retrieval-extinction effect are based upon updating of the original CS-fear memory, or a facilitation of extinction that leads to a stronger inhibitory CS-no fear memory. We are of the opinion that comparison of the molecular pathways required for reconsolidation—especially the destabilisation component of this process—with standard extinction and retrieval-extinction will be highly informative in determining the underlying mechanisms.

As described above, many of the transmitters, receptors and intracellular signalling pathways required for reconsolidation and extinction overlap, perhaps indicating a more general involvement of these molecules in permitting plasticity. However, there are a few differences that could be highly informative in differentiating between the reconsolidation-updating and extinction-facilitation accounts of the attenuation of fear memory following the retrieval-extinction procedure. We would suggest that the requirement for GluN2B-NMDAR-mediated signalling in the amygdala for memory destabilisation could help to distinguish the two accounts, as could the extinction-specific increase in calcineurin expression in the amygdala. If retrieval-extinction recruits reconsolidation-update mechanisms, then it should require GluN2B-NMDAR activity to induce destabilisation, and it should not lead to increases in amygdala calcineurin expression. To date, these predictions have not been tested. It is of note that the retrieval-extinction procedure appears to require reconsolidation-update mechanisms within the hippocampus, being dependent on LVGCCs (Flavell et al. 2011). However, it remains to be determined whether this instead reflects a mechanism by which the contextual specificity of extinction may be reduced.

It is worth reiterating that the attenuation of fear memory observed following retrieval-extinction itself is fragile, with approximately one-third of replication attempts failing (Kindt and Soeter 2013; Goode et al. 2017; Luyten and Beckers 2017) or producing data that are inconsistent with the reconsolidation-update account (Chan et al. 2010). However, other studies have indicated that the mechanism underlying retrieval-extinction depends upon prediction error (Piñeyro et al. 2013) or produces molecular changes consistent with the reconsolidation-update account (Clem and Huganir 2010). A potential explanation for these apparent discrepancies in the literature may be that minor differences in protocols may lead to different mechanisms being engaged (i.e. reconsolidation-update or extinction-facilitation), but with the same behavioural output of reduced recovery of fear. This possibility—that the same reduction in fear behaviour could be produced by two different mnemonic mechanisms, depending on either reconsolidation-update or facilitated extinction—further underlines the importance of referring to molecular evidence to distinguish the underlying mechanism in individual studies.

The clinical value of the retrieval-extinction procedure is not diminished by the lack of consensus over its underlying mechanism, as the marked attenuation of fear observed after this procedure would be highly beneficial for the treatment of anxiety disorders. However, attempts to optimise the retrieval-extinction procedure—through pharmacological enhancement or otherwise—will have a much greater likelihood of success if the mechanism(s) producing the attenuation of fear are understood.

## Abbreviations

DA: Dopamine
Glut: Glutamate
GABA: Gamma-aminobutyric acid
NA: Noradrenaline
mPFC: medial prefrontal cortex
BLA: Basolateral amygdala
PEPA: 4-[2-(phenylsulfonylamino)ethylthio]-2,6-difluoro-phenoxyacetamide
AMPA: α-amino-3-hydroxy-5-methy-4-isoxazole propionate
NMDA: *N*-methyl-d-aspartate
KARs: kainic acid receptors
CNQX: 6-cyano-7-nitroquinoxaline-2,3-dione
NBQX: 1,2,3,4-tetrahydro-6-nitro-2,3-dioxo-benzo[f]quinoxaline-7-sulfonamide
GlutR: Glutamate receptor
CP-AMPAR: Calcium permeable AMPAR
NASPM: 1-naphthylacetyl spermine
LVGCCs: L-type voltage-gated calcium channels
AIDA: 1-aminoindan-1,5-dicarboxylic acid)
DHPG: (R.S.)-3,5-dihydroxyphenylglycine
AMN082: *N*,*N*0-dibenzyhydryl-ethane-1,2-diamine dihydrochloride
APV: 2-Amino-5-phosphonovalerate
CPP: 3-(2-carboxypiperazin-4-yl)propyl-1-phosphonic acid
MPEP: 2-Methyl-6-(phenylethynyl)pyridine
cAMP: adenosine 3′,5′-cyclic monophosphate
H7: 1-(5%-isoquinolinesulfonyl)-2-methylpiperazine
